# Impact of Reviewing Procedure With Visual Gaze Patterns on Improving Endoscopic Submucosal Dissection Skills

**DOI:** 10.1002/jgh3.70193

**Published:** 2025-05-30

**Authors:** Huy Thanh Dang, Fumiaki Ishibashi, Kosuke Okusa, Kentaro Mochida, Takao Tonishi, Sho Suzuki

**Affiliations:** ^1^ Department of Gastroenterology International University of Health and Welfare Narita Hospital Chiba Japan; ^2^ Department of Gastroenterology International University of Health and Welfare Ichikawa Hospital Chiba Japan; ^3^ Department of Business Data Science Faculty of Science and Engineering, Chuo University Tokyo Japan

**Keywords:** endoscopic submucosal dissection, eye‐tracking, training, visual gaze pattern

## Abstract

**Aims:**

No previous studies have reported on whether tracking and reviewing physicians' gaze patterns affect the endoscopic submucosal dissection (ESD) training process. This study investigated differences in physicians' gaze patterns during ESD and assessed how reviewing their procedure afterward impacted ESD skills.

**Methods and Results:**

The gazing points of three trainees during mucosal incision, submucosal dissection, and hemostasis were captured and recorded using the eye‐tracking device. Fifteen short video clips were created from six recorded videos. Three trainees and two expert endoscopists later reviewed these video clips. Key outcomes included: (1) time spent gazing at the appropriate mucosal incision direction, (2) time spent gazing at the appropriate submucosal dissection line, and (3) time required to identify bleeding points. During video review, the trainees spent significantly more time fixating on the appropriate mucosal incision direction than during live performance (24.9 s vs. 6.4 s, *p* < 0.01). However, this was still shorter than expert reviewers (28.4 s, *p* < 0.01). Similarly, the trainees spent more time observing the appropriate submucosal dissection line during review than in real‐time (12.9 s vs. 4.8 s, *p* < 0.05), with no significant difference compared to the experts (14.6 s, *p* = 0.66). However, there was no significant difference in time to identify the bleeding point between review and real‐time performance (9.3 s vs. 11.4 s, *p* = 1.00).

**Conclusion:**

This pilot study suggests that video‐based review with eye‐tracking feedback may help trainees adopt expert‐like visual strategies during ESD, potentially enhancing procedural performance.

## 1 Introduction

Analyzing the visual gaze patterns of endoscopists using eye‐tracking technology is an evolving field of research [1]. Several studies have assessed endoscopists' visual gaze patterns during screening. Gaze analysis suggested a significant correlation between a higher percentage of peripheral gaze fixation and a higher lesion detection rate [2, 3]. During esophagogastroduodenoscopy screening, expert endoscopists spend more time in blind spots such as the greater curvature of the upper part, posterior wall of the body, and lesser curvature of the antrum [4]. Eye tracking technology has also revealed differences in visual gaze patterns between expert and trainee surgeons during various surgeries [5, 6, 7]. In addition, a novel training method that focuses on modifying gaze patterns was shown to be more effective than traditional technical training methods, suggesting the implications of the gaze training method in expediting the learning curves of trainee surgeons [8, 9, 10, 11, 12].

Endoscopic submucosal dissection (ESD), a minimally invasive curative treatment for early gastrointestinal cancer, is technically challenging and has a long learning curve, particularly in regions with an untutored prevalence‐based approach [13]. As the learning curve differs between trainees with and without expert supervision, trainee endoscopists in regions where few ESD experts are available have disadvantages, which prevent the spread of ESD worldwide [14, 15].

Technical hurdles during ESD involve injection, mucosal incision, trimming, submucosal dissection, and hemostasis. Hemorrhage can occur at any time during these steps, and hemostasis can be challenging and stressful, particularly for trainee endoscopists. Furthermore, failure to maintain appropriate gaze points during mucosal incision or submucosal dissection may result in incision into the lesion or injury to the muscle layer. The keys to improving the learning curve are good hemostasis techniques and sufficient skills for mucosal incision and submucosal dissection [16, 17]. Therefore, it is necessary to develop an ESD training method that utilizes expert instructions, even when they are not present during the real‐time procedure, to optimally improve the mentioned skills, expedite the learning process, and promote permeation worldwide. However, no study has evaluated the trainees' gaze patterns during ESD or used them for training.

This study aimed to determine the difference in trainees' gaze patterns while reviewing their ESD videos compared to those during ESD and establish an ESD training method using eye‐tracking technology.

## 2 Materials and Methods

### 2.1 Study Design

This retrospective study was conducted between March 2024 and November 2024. The study protocol was approved by the Ethics Review Board on February 27, 2024 (approval number: 23‐Ic‐008). Written informed consent was not obtained individually because of the retrospective design; however, the study plan was posted on the hospital's website, and patients who did not wish to participate in the study were excluded.

### 2.2 Eye‐Tracking System

**FIGURE 1:**
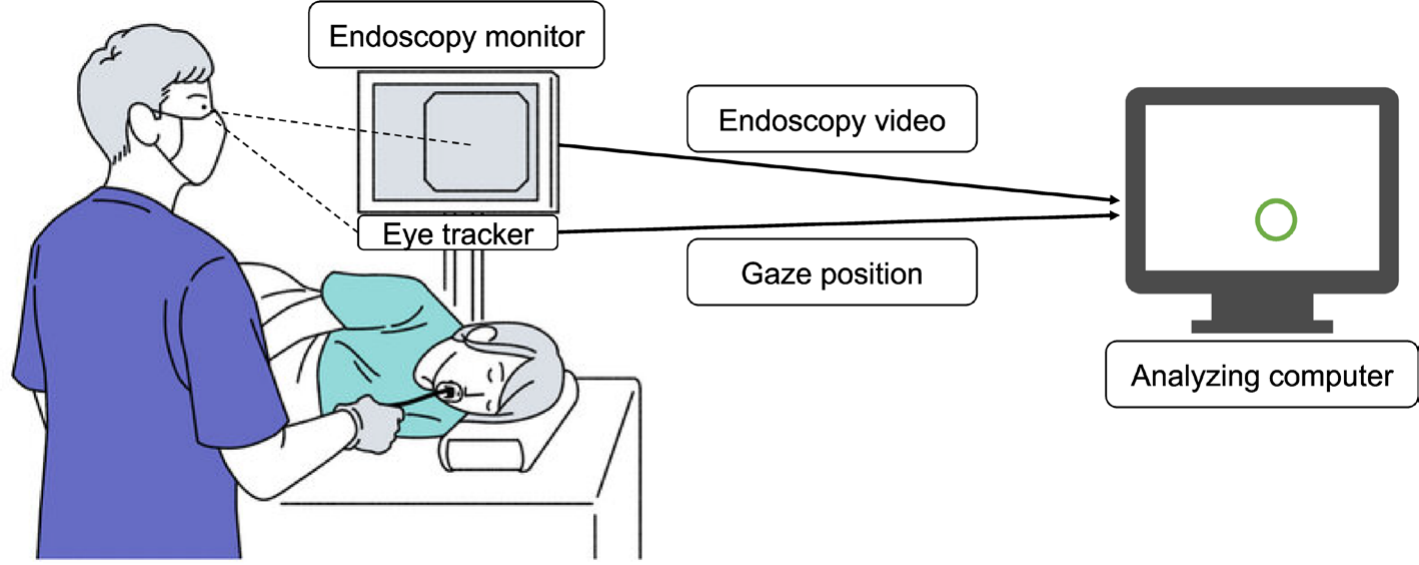
The eye tracking system. A screen‐based eye tracker was attached to the endoscopy screen. Endoscopist's gaze position was captured by the eye tracker and the coordinate information was sent to the analyzing computer. Endoscopy video information was also sent to the analyzing computer and synchronized to synthesis the video with gaze position. Endoscopist's gaze position was described as a green circle in the video.

The screen‐based eye tracker, Tobii Pro Spark (Tobii, Stockholm, Sweden), was directly attached to a 27‐in. endoscopy monitor to capture the gaze position of the operator while performing ESD (Figure 1). The signal from the eye tracker was sent to the analyzing computer, which simultaneously received the video information from the endoscopy light source. The coordinate information of the gaze points was obtained every 30 ms and synchronized with the endoscopy video information on the computer. The dedicated algorithm that we developed previously using Python software could generate an ESD video with tracked gaze positions [18]. The gaze position was overlaid and displayed in the ESD video as a green circle of 30 pixels in size. For the review process, the same eye tracker was attached to a 16‐in. laptop computer screen to capture the reviewer's gaze positions. The operator's gaze position was calibrated before each examination was started. The reviewer's gaze position was also calibrated before the review process. The eye tracker used had a high accuracy of 0.26 root mean square for supplementing eye position.

### 2.3 ESD Procedure With Recording Eye Gaze Positions

Three trainee endoscopists performed gastric ESDs. They had experienced fewer than 10 gastric ESD cases before performing ESDs in this study. The operators stood 1.5 m in front of the monitor to capture their eye movements and record their gaze positions using an eye tracker. The gaze points indicated by the green circle were not displayed on the endoscopy monitor during ESD; therefore, the trainee operators were not aware of their gaze positions. The endoscope systems EG‐760R and EG‐840 T with an ELUXEO 7000 SYSTEM (Fujifilm Co., Tokyo, Japan) were used. An electrosurgical knife (ORISE ProKnife 2.0 mm; Boston Scientific Co., Boston, MA, USA), hemostatic forceps (RAICHO2; Kaneka Medix Co., Osaka, Japan), and a high‐frequency electrosurgical unit (VIO300D; Erbe Elektromedizin GmbH, Marietta, GA, USA) were used.

### 2.4 Reviewing the ESD Video and Re‐Recording Eye Gaze Positions

**FIGURE 2:**
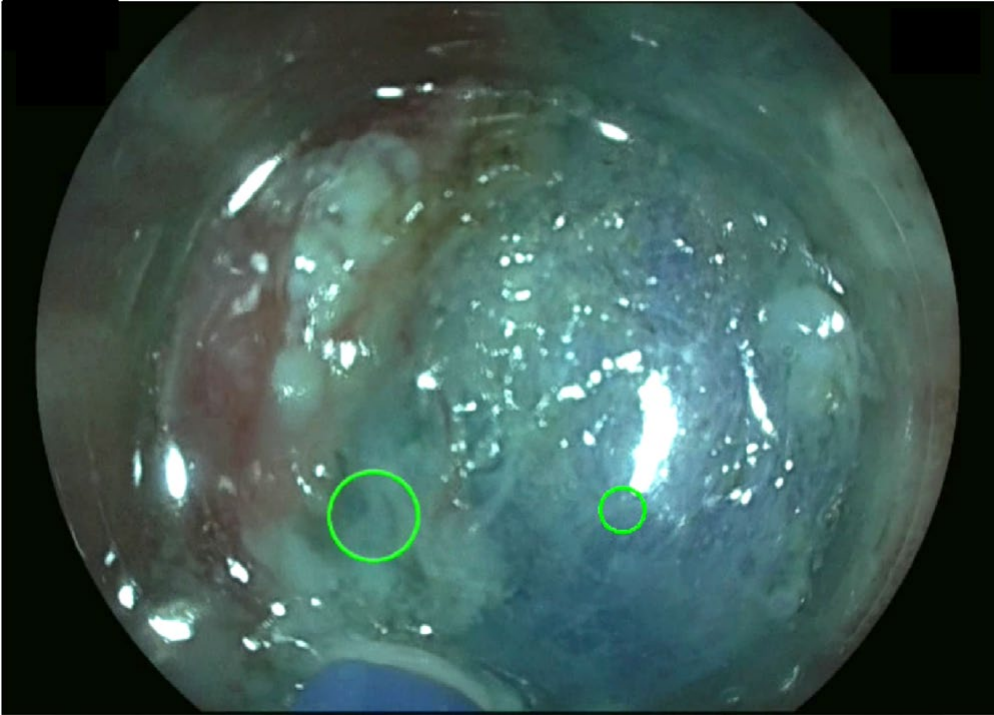
Recording reviewer's gaze position onto the ESD video with operator's gaze position. Large green circle indicates operator's gaze position whereas small green circle indicates reviewer's gaze position.

Videos of gastric ESD performed by trainee endoscopists with their gaze positions displayed were edited to create short clips of mucosal incision, submucosal dissection, and hemostasis. The mucosal incision and submucosal dissection clips were at least 20 s long, during which the tip of the knife was in motion. The clips of hemostasis were edited from the moment the bleeding started until it was confirmed that the bleeding had been stopped by coagulation. The three trainees who performed the ESD and two experts reviewed the videos while their gaze positions were being recorded (Figure 2, Video S1). The reviewers viewed their videos in the monitor room, which was separate from the endoscopy room, one person at a time. The videos were de‐identified and presented in a consistent format to minimize variability. The review results were concealed from the other reviewers to ensure a fair review process. Additionally, the videos were reviewed at least 3 months after the ESD to prevent possible memory bias.

### 2.5 Outcomes

The visual gaze patterns were quantified and compared among the trainees during ESD, the trainees during review, and the experts during review for the following three outcomes: (1) the time spent gazing at appropriate mucosal incision direction, (2) the time spent gazing at appropriate submucosal dissection line, and (3) the time required to identify bleeding points during the hemostasis procedure. The time required to identify bleeding points was defined as the time from the start of bleeding to the endoscopist's fixed gaze position at the bleeding point. The time spent gazing at appropriate mucosal incision direction was defined as the time when the gaze position was away from the tip of the knife and headed toward the marking point of the lesion at an appropriate distance to maintain the margin (Figure S1). Gazing at the incision direction away from the knife tip was assumed to be important for maintaining an appropriate distance from the circumferential marking points of the lesion. An appropriate submucosal dissection line was defined as the line between the middle of the submucosa during the submucosal dissection phase, whereas the dissection line was determined to be inappropriate when the gaze position coincided with either the mucosal or muscle layer (Figure S2).

### 2.6 Statistical Analyses

The continuous variables were checked for normality using the Shapiro–Wilk test. Since the test confirmed that all datasets used in this study did not follow a normal distribution, the Mann–Whitney U test was employed to compare the differences among trainees during ESD, trainees in review, and experts in review. Multiple comparison correction was performed for multiple group comparisons using Bonferroni's method. R software version 4.0.4 was used for statistical analyses. Statistical significance was set at *p* < 0.05.

## 3 Results

### 3.1 Characteristics of ESD and Video Clips for Review

Six ESD procedures performed by three trainees were used to create videos. All lesions were small and located in the middle of the lower body of the stomach (Table 1). The 84 candidate video clips were created from 6 full‐length ESD videos. After excluding 69 short clips deemed unsuitable for review, 15 short clips (5 short clips for hemostasis, 5 clips for mucosal incision, and 5 clips for submucosal dissection) were included in the review and analysis. The average clip duration was 25.8 ± 2.7 s for the hemostasis, 30.4 ± 2.5 s for the mucosal incision, and 29.2 ± 2.9 s for the submucosal dissection. For submucosal dissection, the average time for the tip of the knife to touch the mucosa was 17.0 ± 3.4 s.

**TABLE 1:** Lesion characteristics of ESD cases included in the study.

	Location	Size	Type	Pathology
Case 1	L, Less	10 mm	0‐IIa	tub1, pT1a(M)
Case 2	L, Less	10 mm	0‐IIc	tub1, pT1a(M)
Case 3	L, Gre	8 mm	0‐IIa + IIc	tub1, pT1a(M)
Case 4	L, Less	10 mm	0‐IIc	tub1, pT1a(M)
Case 5	M, Less	8 mm	0‐IIa	tub1, pT1a(M)
Case 6	L, Ant	6 mm	0‐IIc	tub1, pT1a(M)

Abbreviations: Ant, anterior wall; ESD, endoscopic submucosal dissection; Gre, greater curvature; L, lower part of the stomach; Less, lesser curvature; M, middle part of the stomach.

### 3.2 Study Outcomes of Changing Gaze Pattern

#### 3.2.1 Mucosal Incision Direction

**FIGURE 3:**
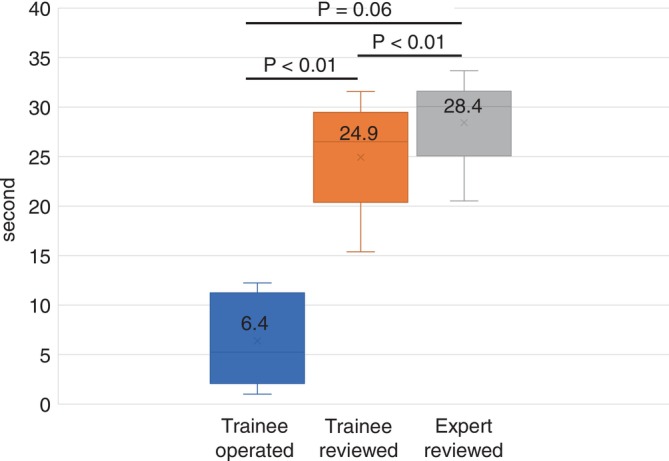
The time of gazing at the incision direction during mucosal incision. The time when the trainees reviewed was significantly longer than that when the trainees operated (24.9 s vs. 6.4 s, *p* < 0.01) but did not reach that of the expert reviewers (24.9 s vs. 28.4 s, *p* = 0.04).

The time spent gazing at the appropriate mucosal incision direction when the trainees reviewed was significantly longer than that when the trainees operated (24.9 s vs. 6.4 s, *p* < 0.01) but shorter than that of the expert reviewers (24.9 s vs. 28.4 s, *p* < 0.01) (Figure 3). The time of gazing at the knife tip instead of the incision direction of the trainee reviewers was significantly shorter than that of the trainee operators (5.5 s vs. 23.8 s, *p* < 0.01) but significantly longer than that of the expert reviewers (5.5 s vs. 2.1 s, *p* < 0.01).

#### 3.2.2 Submucosal Dissection Line

**FIGURE 4:**
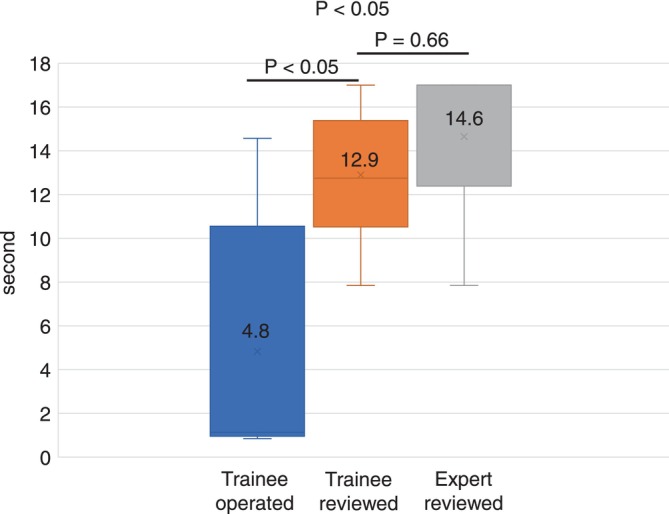
The time gazing at the appropriate dissection line during submucosal dissection. The time when the trainee reviewed was significantly longer than that when the trainee operated (12.9 s vs. 4.8 s, *p* = 0.01) and reached the level in the expert review (12.9 s vs. 14.6 s, *p* = 0.10).

The time spent gazing at appropriate submucosal cutting line when the trainee reviewed was significantly longer than that when the trainee operated (12.9 s vs. 4.8 s, *p* < 0.05), and it was not significantly different from that of expert reviewers (12.9 s vs. 14.6 s, *p* = 0.66) (Figure 4). The time spent gazing at the exterior of the submucosal layer when the trainee reviewed was significantly shorter than that when the trainee operated (3.0 s vs. 10.8 s, *p* = 0.04), but similar to that when the expert reviewed (3.0 s vs. 2.3 s, *p* = 0.51). There was no significant difference in the time gazing at the muscle layers when the trainee reviewed and operated (1.0 s vs. 1.0 s, *p* = 1.00), whereas the time gazing at the muscle layers when the expert reviewed was significantly shorter than that when the trainee reviewed and operated (0 s vs. 1.0 s, *p* = 0.03; 0 s vs. 1.0 s, *p* < 0.01).

#### 3.2.3 Hemostasis: Identifying Bleeding Point

**FIGURE 5:**
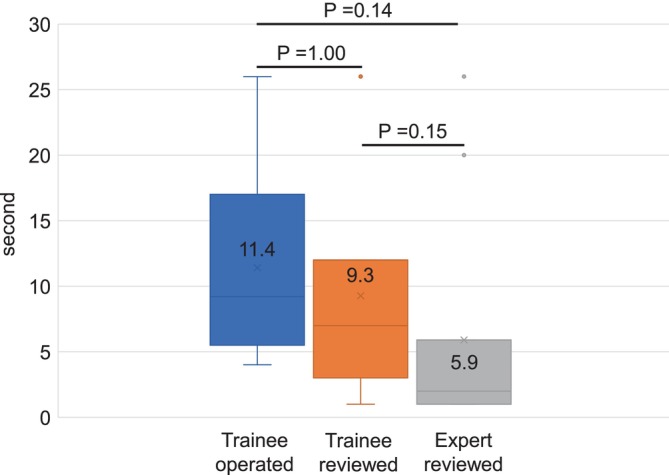
The time required for identifying the source of bleeding during hemostasis. The time when the trainee reviewed was shorter than that when the trainee operated but not significant (9.3 s vs. 11.4 s, *p* = 0.40), whereas it was longer than that when the expert reviewed (9.3 s vs. 5.9 s, *p* = 0.05).

There was no significant difference in the time to identify the bleeding source between when the trainees reviewed the procedure and when they performed it (9.3 s vs. 11.4 s, *p* = 1.00). However, there was no significant difference in the time taken to identify the bleeding source between when the trainees reviewed the procedures and when the experts reviewed them (9.3 s vs. 5.9 s, *p* = 0.15) (Figure 5).

## 4 Discussion

ESD is technically challenging and has a long learning curve, particularly in regions with an untutored, prevalence‐based approach [13]. To date, there have been no reports on the application of eye tracking technology to ESD training. In this study, we successfully recorded and quantified the visual gaze patterns of endoscopists during and while reviewing ESD using eye‐tracking technology. We examined whether trainees could adopt the ideal gaze pattern by focusing only on gazing when reviewing videos after ESD. The time the trainee reviewers spent gazing at the incision direction and the appropriate submucosal dissection line was also significantly longer. At our institution, all trainees receive a lecture from an expert on the appropriate gaze position while assisting with the expert's procedures before actually performing ESD. Therefore, the appropriate incision direction and cutting line, which were set as outcomes in this study, were acquired through knowledge transfer from the experts to the trainees. The reason why the trainees were unable to achieve proper eye position during ESD but could achieve it in a calm environment may be due to distractions caused by various environmental factors, such as having to manipulate the scope and knife, checking the patient's body movements, and monitoring the sound on the monitor.

This study showed that eye position interventions have the potential to accelerate ESD education. The improvement of the visual gaze patterns is expected to help shorten total procedure times. Notably, maintaining an appropriate mucosal incision direction contributes to minimizing trajectory error, while correcting the visual gaze pattern during submucosal dissection may prevent complications such as bleeding and perforation. The trainee's visual gaze pattern during mucosal cutting and submucosal dissection was quickly corrected during ESD procedures, however, this was not improved during hemostasis. These results suggest that it may take time for trainees to understand the ideal gaze position for achieving hemostasis when acquiring ESD skills. The trainees' understanding of the ideal gaze pattern varies across each phase of ESD and necessitates distinct pedagogical approaches. Specifically, it is helpful to instruct the trainee to widen the field of view during all phases of ESD without rushing. However, for quick identification of the bleeding point, widening the field of view only is insufficient; therefore, alternative educational approaches, such as improving trainees understanding of bleeding point indicative signs, are also necessary.

During mucosal incision, the incision should be made at a distance from the lesion to maintain a safety margin, which requires endoscopists to look at the incision direction to minimize trajectory error [17]. In addition, unless proper pressure is applied to the knife, the blood vessels may not coagulate properly, resulting in hemorrhage [17]. In this study, trainee endoscopists tended to look at the tip of the knife during mucosal incision rather than in the incision direction, which can be explained by the narrowing of the field of vision during mucosal resection. The fact that the trainees were able to gaze at the incision direction during the review but not during the procedure indicates that although they understood the ideal gaze pattern, they were not able to put it into practice during the procedure. Therefore, encouraging trainees to concentrate on creating ideal gaze patterns is suggested to lead to the early acquisition of ESD techniques.

The appropriate dissection depth was in the middle of the submucosa [17]. Large vessels in the gastrointestinal luminal wall penetrate the muscle layer vertically and branch out into the superficial part of the submucosa; therefore, maintaining the appropriate dissection depth can prevent massive hemorrhage [19]. In addition, it is important to determine the appropriate depth to maintain safety margins from both the lesion and the muscle layer. In this study, trainee operators tended to gaze at and dissect the superficial part of the submucosa, whereas trainee reviewers tended to gaze at and dissect the appropriate submucosal dissection lines. This may be because the trainees were afraid of perforation due to muscle damage and tended to look at areas farther away from the muscle layer than necessary. The fact that trainee reviewers spent more time gazing at the appropriate submucosal dissection line suggests that eye gaze review can help trainees visually understand their mistakes and facilitate improvement in their ESD skills.

Intraoperative hemorrhage can occur at any time during submucosal injection, incision, or dissection. The identification of bleeding points and mastering hemostasis techniques are crucial to ensure a safe and time‐efficient procedure. Eye‐tracking technology offers an objective method to confirm the time required for bleeding point identification. In this study, the time required by both trainee operators and reviewers to identify bleeding points was longer than that required by the expert reviewers. The fact that it took the trainees time to identify the bleeding point even in a calm environment during the review suggested that, to acquire hemostasis skills, it was necessary to first train the trainees to quickly identify the bleeding point using video materials rather than during real ESD.

Several studies have compared the differences in visual gaze patterns between expert and trainee surgeons during various surgeries and suggested that experts spent significantly more time fixated on the target locations than trainees [5, 6, 7]. In addition, a novel training method focusing on gaze training was shown to be more effective than traditional technical training methods, suggesting the implications of the gaze training method in expediting the learning curves of trainee surgeons [8, 9, 10, 11, 12]. Gaze training reportedly improves surgical training [8, 9]. Several reports have also suggested that experts' gaze can be used to make teaching videos [10], and surgery training tools utilizing eye‐tracking technology to provide visual instruction can lead to an improvement in the performance of trainees [11, 12]. These studies and ours suggest that eye‐gaze training could be an effective training method to expedite the learning curve of trainees. Collectively, these findings indicate that training based on the trainee's gaze position may accelerate the acquisition of other endoscopic procedure skills, including endoscopic retrograde cholangiopancreatography, endoscopic ultrasound, and robotic surgery.

This study has several limitations. First, since this study was conducted retrospectively with only three trainees at a single facility, it involved selection bias and other potential biases. Additionally, the statistical power to detect differences before and after education is likely to be low. Second, the eye gazes of the expert and trainee while reviewing the video clip may have been affected by the eye gaze positions of the trainee operators. In future studies, using original video clips with and without the trainee endoscopists' gaze position would better clarify the impact of the operator's gaze position on reviewers' gaze behavior. Third, the potential confounders, including the level of fatigue, variability in lesion characteristics, or prior exposure to ESD techniques, might influence the results of initial procedures and the review process. Fourth, although this study set the interval between the ESD operation and reviewing video at more than 3 months, memory bias could not be eliminated. These limitations inevitably reduce the study's generalizability. Nevertheless, this study established a method for analyzing visual gaze patterns, which may yield more findings when applied to a larger number of trainees across multiple facilities. This study is positioned as a pilot study to provide a basis for conducting a future large‐scale study.

## 5 Conclusions

This study showed that the trainees' visual gaze patterns during ESD changed after reviewing their procedure videos. In particular, the time spent gazing at appropriate mucosal incision direction and submucosal dissection line was significantly improved in the reviewed trainee compared with the operated trainee. Despite being a pilot study with limited generalizability, this result suggests that reviewing procedure videos may help improve ESD skills in terms of physicians' visual gaze patterns. New educational approaches considering physicians' visual gaze patterns are warranted in the clinical practice of endoscopy.

## Conflicts of Interest

Fumiaki Ishibashi received honoraria for lectures from FUJIFILM Corporation. Sho Suzuki received honoraria for lectures from FUJIFILM Corporation and Olympus Corporation. The other authors declare no conflicts of interest.

## Supporting information


**Figure S1.** Definition of an appropriate mucosal incision direction. The appropriate direction was defined as the direction away from the tip of the knife and headed toward the marking point of the lesion at an appropriate distance to maintain the margin. The white circle indicates the area covering the tip of the knife, which is considered an inappropriate gaze position. In contrast, the white shaded fan‐shaped area was considered the appropriate gaze position.

**Figure S2.** Definition of an appropriate submucosal dissection line. The appropriate line was defined as the line between the middle of the submucosa during the submucosal dissection phase (between the two white lines), whereas the dissection line was determined to be inappropriate when the gaze position coincided with either the mucosal or muscle layer (outside the two white lines).

**Video S1.** The eye‐gaze position of the trainee operated and reviewed during submucosal dissection. A big green circle indicates the eye‐gaze position of the trainee operated, whereas a small green circle indicates that of the trainee reviewed.
