# Supplementary figures and images for: Impact of Reviewing Procedure With Visual Gaze Patterns on Improving Endoscopic Submucosal Dissection Skills
Source: JGH Open. 2025 May 30;9(6):e70193. doi: 10.1002/jgh3.70193 (PMC12123096; doi:10.1002/jgh3.70193)

**Supplementary Fig. 1**

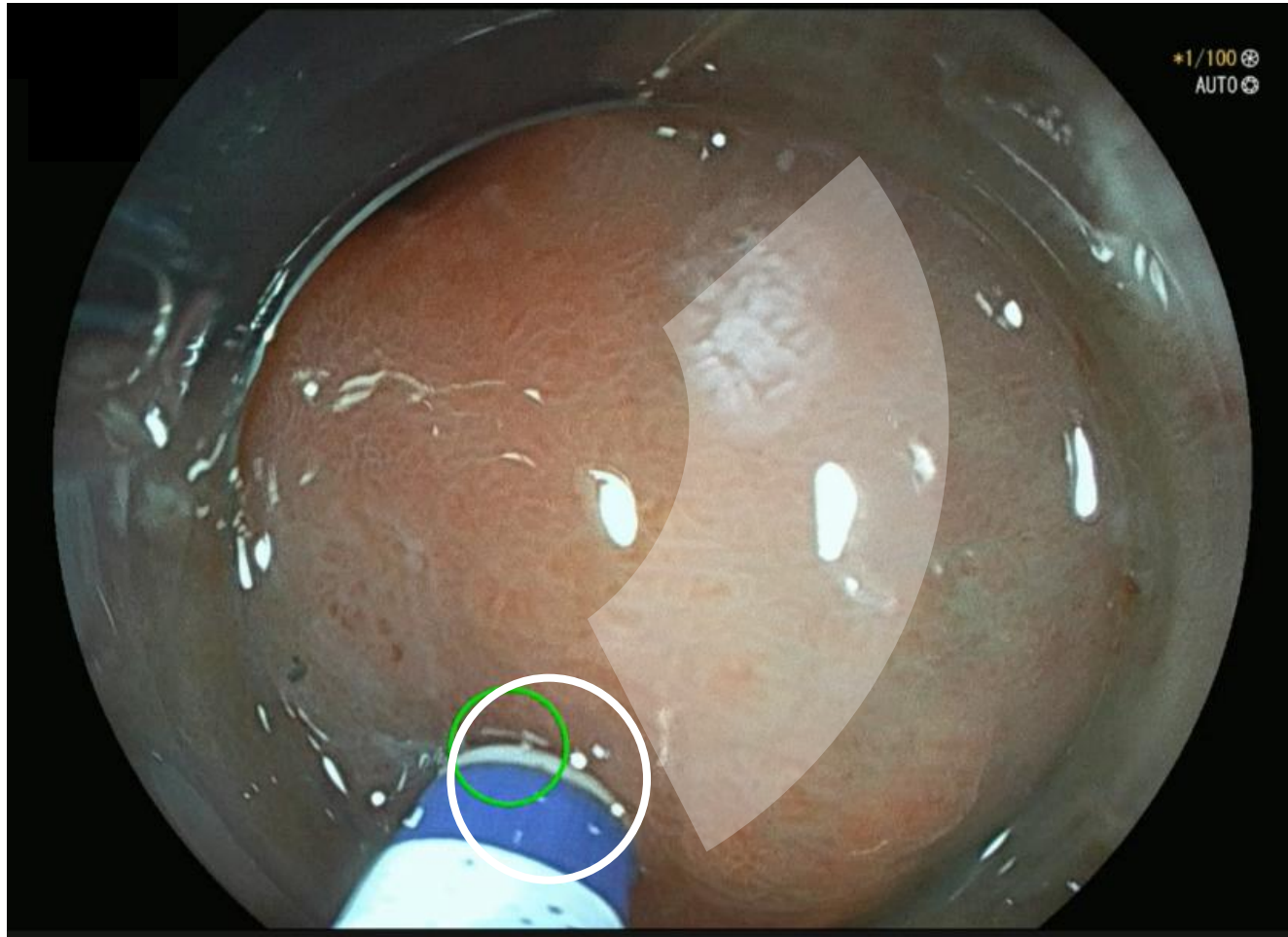

Supplement: Supplementary file 1 — Figure S1. Definition of an appropriate mucosal incision direction. The appropriate direction was defined as the direction away from the tip of the knife and headed toward the marking point of the lesion at an appropriate distance to maintain the margin. The white circle indicates the area covering the tip of the knife, which is considered an inappropriate gaze position. In contrast, the white shaded fan‐shaped area was considered the appropriate gaze position. [file JGH3-9-e70193-s003.pdf]

Supplementary Fig. 2

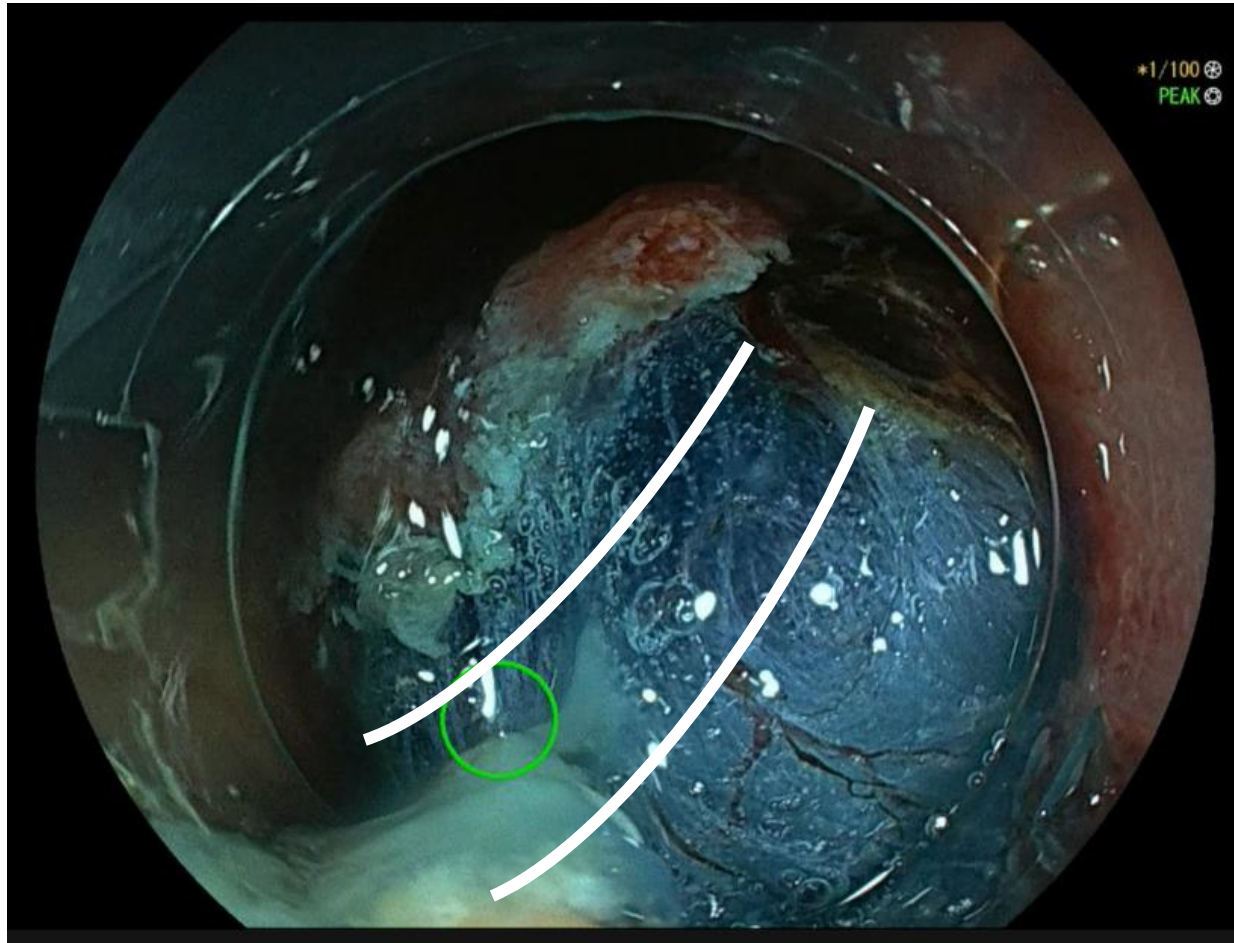

Supplement: Supplementary file 2 — Figure S2. Definition of an appropriate submucosal dissection line. The appropriate line was defined as the line between the middle of the submucosa during the submucosal dissection phase (between the two white lines), whereas the dissection line was determined to be inappropriate when the gaze position coincided with either the mucosal or muscle layer (outside the two white lines). [file JGH3-9-e70193-s001.pdf]
